# IFN-γ Mediates the Rejection of Haematopoietic Stem Cells in IFN-γR1-Deficient Hosts

**DOI:** 10.1371/journal.pmed.0050026

**Published:** 2008-01-29

**Authors:** Martin Rottman, Claire Soudais, Guillaume Vogt, Laurent Renia, Jean-François Emile, Hélène Decaluwe, Jean-Louis Gaillard, Jean-Laurent Casanova

**Affiliations:** 1 Laboratoire de Génétique Humaine des Maladies Infectieuses, INSERM, U550, Paris, France; 2 Université Paris René Descartes, Faculté de Médecine Necker-Enfants Malades, Paris, France; 3 Hôpital Raymond Poincaré, Faculté de Médecine Paris-Ile de France-Ouest, UPRES Sud, EA3647, Laboratoire de Microbiologie, Garches, France; 4 Institut Cochin, INSERM, U567, Paris, France; 5 CNRS, UMR8104, Paris, France; 6 Université René Descartes, Hôpital Cochin, Paris, France; 7 Hôpital Ambroise Paré, Laboratoire d'Anatomo-Pathologie, Boulogne, France; 8 Unité d'Immunologie et Hématologie Pédiatriques, Hôpital Necker-Enfants Malades, Paris, France; Institute of Child Health, United Kingdom

## Abstract

**Background:**

Interferon-γ receptor 1 (IFN-γR1) deficiency is a life-threatening inherited disorder, conferring predisposition to mycobacterial diseases. Haematopoietic stem cell transplantation (HSCT) is the only curative treatment available, but is hampered by a very high rate of graft rejection, even with intra-familial HLA-identical transplants. This high rejection rate is not seen in any other congenital disorders and remains unexplained. We studied the underlying mechanism in a mouse model of HSCT for IFN-γR1 deficiency.

**Methods and Findings:**

We demonstrated that HSCT with cells from a syngenic C57BL/6 *Ifngr1* ^+/+^ donor engrafted well and restored anti-mycobacterial immunity in naive, non-infected C57BL/6 *Ifngr1* ^−/−^ recipients. However, *Ifngr1* ^−/−^ mice previously infected with Mycobacterium bovis bacillus Calmette-Guérin (BCG) rejected HSCT. Like infected IFN-γR1-deficient humans, infected *Ifngr1* ^−/−^ mice displayed very high serum IFN-γ levels before HSCT. The administration of a recombinant IFN-γ-expressing AAV vector to *Ifngr1* ^−/−^ naive recipients also resulted in HSCT graft rejection. Transplantation was successful in *Ifngr1* ^−/−^ × *Ifng* ^−/−^ double-mutant mice, even after BCG infection. Finally, efficient antibody-mediated IFN-γ depletion in infected *Ifngr1* ^−/−^ mice in vivo allowed subsequent engraftment.

**Conclusions:**

High serum IFN-γ concentration is both necessary and sufficient for graft rejection in IFN-γR1-deficient mice, inhibiting the development of heterologous, IFN-γR1-expressing, haematopoietic cell lineages. These results confirm that IFN-γ is an anti-haematopoietic cytokine in vivo. They also pave the way for HSCT management in IFN-*γ*R1-deficient patients through IFN-γ depletion from the blood. They further raise the possibility that depleting IFN-γ may improve engraftment in other settings, such as HSCT from a haplo-identical or unrelated donor.

## Abstract 2

Claire Soudais and colleagues investigated the mechanism of rejection of hematopoietic stem cell transplants in patients with interferon-gamma receptor 1 (IFN-γR1) deficiency and show that IFN-γ is an anti-hematopoietic cytokine in vivo.

## Editors' Summary

**Background.:**

Normally, the body's immune system efficiently recognizes and kills bacteria and viruses, but in some rare inherited disorders (“primary immunodeficiencies”) part of the immune system works poorly or is missing. This leaves affected individuals susceptible to infections. People with one of these disorders—interferon-gamma receptor 1 (IFN- γR1) deficiency—are very susceptible to infections with mycobacteria. Except for Mycobacterium tuberculosis and M. leprae (which cause tuberculosis and leprosy, respectively), mycobacteria rarely cause human disease. However, most people with IFN-γR1 deficiency die during childhood from multiple, widespread mycobacterial infections, because IFN-γR1 deficiency disables a specific part of their immune system. When most bacteria enter the body, immune system cells called macrophages engulf and kill them, but mycobacteria actually multiply inside macrophages. This infection stimulates lymphocytes and other immune system cells to release IFN-γ, which binds to IFN-γR1 on uninfected macrophages, activates them, and recruits them to the infection site. Here, they form a “granuloma,” a mass of macrophages and activated lymphocytes that “walls off” the infection. Granuloma formation does not occur in patients with IFN-γR1 deficiency, so mycobacteria (including the usually benign tuberculosis vaccination strain M. bovis BCG) spread throughout the body with disastrous consequences.

**Why Was This Study Done?:**

The only effective treatment for patients with IFN-γR1 deficiency is hematopoietic stem cell transplantation (HSCT). HSCs are the source of all the immune system cells, so transplantation of HSCs from a donor with a normal *IFNGR1* gene can provide a patient who has IFN-γR1 deficiency with a new immune system that can combat mycobacterial infections. Unfortunately, in this particular immune deficiency, the new HSCs cannot engraft, even when the patient's own immune system is disabled before HSCT by intensive chemotherapy, and when the donor cells come from a close relative and are a good immunological match. In this study, the researchers have investigated why rejection is so common in IFN-γR1 deficiency using a mouse strain called C57BL/6 *Ifngr1* ^−*/*−^ *—*C57BL/6 denotes the genetic background of these mice and *Ifngr1* ^−*/*−^ indicates that, like human patients, these mice make no IFN-γR1.

**What Did the Researchers Do and Find?:**

*Ifngr1* ^−*/*−^ mice, the researchers report, cannot control M. bovis BCG infections and do not form mature granulomas just like human patients with IFN-γR1 deficiency. Wild-type C57BL/6 (*Ifngr1^+/+^*) mice, however, rapidly control M. bovis BCG infections and form mature granulomas. *Ifngr1^+/+^* HSC transplanted into mycobacteria-free *Ifngr1* ^−*/*−^ mice survived well and protected the mice against later mycobacterial challenge but *Ifngr1* ^−*/*−^ mice infected with M. bovis BCG before HSCT rejected the transplanted HSCs. Mycobacteria-infected *Ifngr1* ^−*/*−^ mice and human patients with IFN-γR1 deficiency have blood high levels of IFN-γ. Could this be responsible for HSCT rejection? To find out, the researchers expressed IFN-γ in uninfected *Ifngr1* ^−*/*−^ mice before HSCT. As in infected mice, these grafts failed. Conversely, transplanted HSCs survived when transplanted into *Ifngr1* ^−*/*−^ mice that had been genetically altered to express no IFN-γ, even when these mice were infected with M. bovis BCG before transplantation. Finally, when the researchers used antibodies (proteins made by the immune system that recognize specific molecules) to remove circulating IFN-γ from infected *Ifngr1* ^−*/*−^ mice, HSCT worked well in the animals with the lowest IFN-γ levels.

**What Do These Findings Mean?:**

These findings indicate that in a mouse model of IFN-γR1 deficiency, high circulating IFN-γ concentrations drive the rejection of transplanted HSCs and prevent the development of antimycobacterial immunity, probably by directly killing the transplanted cells and/or stopping them multiplying. They also suggest how HSCT could be improved in patients with IFN-γR1 deficiency although, as with all animal studies, the situation in people might turn out to be very different. Importantly, antibodies that reduce circulating IFN-γ are already being used to treat other human immune diseases, so the clinical use of these antibodies in patients with IFN-γ deficiency before HSCT is feasible. Finally, the researchers speculate that the use of IFN-γ–depleting antibodies might be beneficial in other situations where HSCT often fails such as when a close relative is not available as a donor. However, this possibility will need to be thoroughly tested in mice before human clinical trials can be started.

**Additional Information.:**

Please access these Web sites via the online version of this summary at http://dx.doi.org/10.1371/journal.pmed.0050026.

- General information about primary immunodeficiencies is available from the US National Institute of Child Health and Human Development
- Online Mendelian Inheritance in Man (OMIM) provides information about familial predisposition to mycobacterial disease
- Wikipedia has pages on hematopoietic stem cell transplantation and on interferon-γ (note that Wikipedia is a free online encyclopedia that anyone can edit; available in several languages)
- The Human Genetics of Infectious Diseases Lab focuses on the genetic basis of predicposition or resistance to infectious diseases in humans

## Introduction

Complete deficiency of the ligand-binding chain of the interferon-γ receptor (IFN-γR1) is an autosomal recessive disorder described in 1996 as the first genetic aetiology of the syndrome of Mendelian Susceptibility to Mycobacterial Diseases (“MSMD”: MIM 209950) [1–3]. Causal mutations either abrogate cell surface IFN-γR1 expression [4,5] or prevent IFN-γ recognition due to the production of surface-expressed, non-functional receptors [6]. Both types of IFN-γR1 deficiency result in a complete loss of cellular responses to IFN-γ. This disorder confers a profound and selective susceptibility to weakly virulent mycobacteria, such as Mycobacterium bovis bacillus Calmette Guérin (BCG) vaccines and environmental mycobacteria [7], as reviewed in [3]. Humans are also susceptible to the more virulent M. tuberculosis [7,8]. Other infectious diseases are rare, with the exception of salmonellosis, which has been diagnosed in several patients [9]. Listeriosis and a few viral diseases were each diagnosed in single patients [7,10]. Humans do not produce mature granulomas in response to mycobacteria, and instead display poorly delimited, poorly differentiated, multibacillary tissue lesions [11]. They present with early-onset, disseminated, recurrent, and multiple mycobacterial infections. Most patients die in early childhood, with only one-third surviving to the age of 15 years [7].

The treatment of patients with complete IFN-γR1 deficiency is particularly difficult [3,7]. Antibiotics alone do not achieve permanent clinical remission. Unlike patients with other MSMD aetiologies, those with complete IFN-γR1 (or IFN-γR2) deficiency do not benefit from exogenous IFN-γ administration, owing to the lack of a specific receptor. The remission of mycobacterial infection following the first case of haematopoietic stem cell transplantation (HSCT) in such an individual provided proof-of-principle that IFN-γR1 deficiency is primarily a haematopoietic disorder, despite the ubiquitous expression of IFN-γR1 [12]. However, subsequent attempts revealed a very high rate of primary and secondary rejection in HLA-identical HSCT [13–15]. Nine patients received a total of 12 transplants. Four of these patients died within eight months of transplantation—from mycobacterial disease in two cases—and three individuals rejected the graft. Two of the five patients who survived presented only very low levels of chimerism and no chimerism was observed in a third. One individual had a low-grade infection at the time of the treatment [15]. Only three individuals have remained free from infectious complications [13,15] and are currently healthy, nine, seven, and seven years after HSCT. This rate of HLA-identical graft rejection is by far the highest reported for HSCT to treat primary immunodeficiencies or congenital disorders [16]. These observations indicate that HSCT is potentially curative in IFN-γR1-deficient individuals, but associated with a particularly high and unexplained rate of graft rejection, resulting in unacceptable morbidity and mortality rates.

We used mice selectively deficient in IFN-γ R1 (*Ifngr1^−/−^*), IFN-γ (*Ifng^−/−^*), or both (*Ifngr1^−/−^* × *Ifng^−/−^*) [17,18], as a means of investigating the mechanism of graft rejection in IFN-γR1-deficient patients. These mice are susceptible to several intracellular pathogens [17,19–23]. In particular, *Ifngr1^−/−^* mice are highly susceptible to mycobacteria, such as M. tuberculosis [24,25], M. avium [26] and BCG [27]. *Ifngr1^−/−^* mice die within nine weeks of the intravenous injection of 2 × 10^7^ cells of BCG*,* whereas control mice survive such injections [27]. BCG is also the most common pathogen in IFN-γR1-deficient individuals [7]. On histological examination, these patients have tissue lesions containing large numbers of acid-fast BCG, with small numbers of structurally impaired granulomas and high tissue loads of mycobacteria [4,11]. The same infectious phenotype has been observed in *Ifng^−/−^* mice infected with BCG [18]. Thus, the extreme susceptibility of *Ifngr1^−/−^* mice to mycobacterial infections, including BCG in particular, mimics the human condition, making these mice an ideal model for studies of the mechanisms underlying the HSCT failure observed in IFN-γR1-deficient humans.

## Materials and Methods

### Animals

Specific pathogen-free, *Ifngr1* ^+/+^ C57BL/6 mice were purchased from Charles River (L'Arbresle, France) and used at six to nine weeks of age. Interferon gamma receptor chain 1 (*Ifngr1^−/−^*, stock no. 003288, N10) [17] and interferon gamma (*Ifng^−/−^*, stock no. 002287, N10) [18] mice were purchased from JaxLab and reared at the CDTA (Centre de Distribution, Typage et Archivage animal, Orléans, France). Both *Ifngr1^−/−^* and *Ifng^−/−^* mice were backcrossed onto the C57BL/6 background for more than ten generations. Double knock-out (DKO) mice (*Ifngr1^−/−^* × *Ifng^−/−^*) were generated in a pathogen-free animal facility, by mating homozygous *Ifng^−/−^* mice with *Ifngr1^−/−^* mice. The resulting F1 animals were then intercrossed to generate double homozygous mice, identified by PCR on tail DNA (primers and conditions available from the JaxLab Web site [http://jaxmice.jax.org/strain/002287.html and http://jaxmice.jax.org/strain/003288.html]). All experiments and procedures were performed in accordance with French Ministry of Agriculture regulations for animal experimentation (1987) and the guidelines of our institution's animal welfare committee.

### Haematopoietic Stem Cell Transfer

Recipient mice were subjected to intensive or milder doses (550 rads to 1,200 rads) of body irradiation, using a Cs137 radioactive source (Pasteur Institute). Immune reconstitution was then initiated by the intravenous administration, via a lateral tail vein, of two million total bone marrow cells freshly isolated from donor mice. Briefly, bone marrow cells were flushed into PBS from the femurs and tibias of donor mice. Red blood cells were lysed and the remaining cells were counted before bone marrow cell transfer. After engraftment, mice were kept in a specific-pathogen-free animal facility, in filter-topped cages in an isolation room. All caging procedures and manipulations were carried out in a laminar flow hood. Mice were used for experimental infection eight weeks after HSCT.

### Analysis of Chimerism and Reconstitution

The extent of haematopoietic reconstitution by the donor phenotype was assessed every two weeks, during the eight weeks before experimental infection, using specific antibodies. Immunofluorescence analyses were carried out on whole blood. Briefly, donor bone marrow cells expressed the Ly5.1 marker, whereas recipient leukocytes expressed the Ly5.2 marker. Chimerism was therefore assessed as the percentage of cells expressing the Ly5.1 isotype (engraftment). Antibodies against the following surface antigens (all from BD Bioscience Pharmingen) were used, as FITC or PE conjugates, to evaluate peripheral reconstitution: TCRαβ, B220, Mac-1 and GR1. Blood (25 μl) was first blocked with 24G2 serum, then stained with specific antibodies. Red blood cells were then lysed with PharM Lyse buffer and flow cytometry was carried out on the remaining cells (FACScan, Becton Dickinson).

### Experimental Infection with BCG and Determination of the Number of Colony-Forming Units

BCG strain Pasteur 1173P_2_ was used [28]. BCG was prepared as described elsewhere [29], frozen in aliquots and stored at −80 °C. For each infection experiment, groups of non-irradiated animals were included as positive and negative controls of infection. Bacteria were counted in the spleen and liver, as described elsewhere [29]. In brief, blood samples were taken from six to eight mice for each point. The mice were then killed, and their organs were dissected out, placed in 2 ml screw-cap tubes filled with sterile water and homogenized with a 5 mm stainless steel ball (SKF) using a mini-8 bead-beater (Biospec). Spots (50 μl) of serial five-fold dilutions of this suspension were plated on Middlebrook 7H11 medium supplemented with OADC (Difco). The plates were incubated for 20 d at 37 °C, under a humidified atmosphere containing 5% CO_2_, and colonies were counted using a stereoscopic binocular microscope with a detection threshold of 170 CFU per organ.

### ELISA

Serum was recovered from whole blood samples following coagulation. Serum aliquots were frozen at −20 °C for quantitative sandwich immunoassays for IFN-γ. Mouse ELISA kits from R&D Systems were used, according to the manufacturer's recommendations, in all experiments. The detection limit of the kit was less than 2 pg/ml.

### RNA Purification and Northern Blot Analysis

Tissue samples were lysed in 1 ml of Trizol (Invitrogen) and the resulting suspensions were immediately frozen and stored at −80 °C. Total RNA was extracted with a kit based on the acid phenol-guanidine method, according to the manufacturer's instructions. Total RNA concentration was estimated by spectrophotometry. Total RNA (15 μg in 50% formamide) was blotted onto a nylon membrane. The membrane was incubated for three hours at 80 °C and was then washed in 2×SSC before hybridisation with murine IFN-γ and GAPDH probes generated by PCR (primers available on request) and labelled with ^32^P by random priming. Each autoradiograph was densitometrically scanned and levels of the specific IFN-γ transcripts were normalised with respect to GAPDH transcript levels.

### Preparation and Use of Recombinant Adeno-Associated Vectors and Injection

The mouse IFN-γ cDNA was amplified by PCR from the L929 cell line and inserted into convenient restriction sites in the pGG1 vector. The resulting plasmid was sequenced to check the integrity of the mIFN-γ cDNA. We prepared rAAV-IFN-γ particles, as previously described [30], by a helper-virus-free method involving the triple transfection of HEK 293 cells. Physical particles were estimated by dot plots, and 1.7 × 10^12^ such particles were obtained per ml of preparation [31]. We assessed rAAV-IFN-γ particle function in vivo, by injecting 5 × 10^10^ rAAV particles, in a final volume of 100 μl, into the left gastrocnemius muscle of recipient *Ifngr1^−/−^* mice. Serum samples were recovered at various time points after injection and mIFN-γ was quantified by ELISA. A rAAV vector including the *lacZ* gene was used as negative control.

### Antibody-Mediated Cytokine Neutralisation

Animals were infected with 10^6^ CFU of BCG. Fourteen days later, they received an intraperitoneal injection of a mixture of 0.8 mg of purified rat anti-mouse IFN-γ antibody (clone XMG1 [32]) and 0.8 mg of purified rat anti-mouse IL-12p40 antibody (clone C15–1 [33]). Antibodies were purified from the culture supernatant by ammonium sulphate precipitation and shown to function effectively in a mouse model of *Plasmodium* infection [34]. The control group received an injection of 1.6 mg of irrelevant purified rat IgG antibody (Sigma). In all groups, the injection was repeated on days 15, 17, 20, and 24. Bone marrow from *Ifngr1^+/+^* donors was transferred to the recipient mice 23 d after sublethal body irradiation (550 rad). Serum IFN-γ concentration and chimerism were monitored throughout the experiment.

### Histology

Organs were fixed by immersion for 24 h in 3.7% formaldehyde and were then transferred to 70% ethanol for 24 h to 48 h before embedding in paraffin. Sections (5 μm) were cut on a rotary microtome, stretched in a water bath, mounted on glass slides and stained with haematoxylin-eosin-saffron (HES) or by the Zielh-Neelsen method.

## Results

### *Ifngr1^−/−^* Mice Are Susceptible to BCG Infection

**Figure 1:**
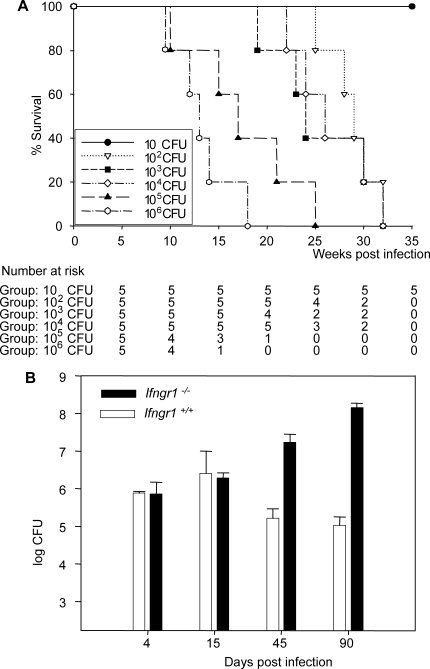
*Ifngr1^−/−^* Mice Are Susceptible to BCG Infection (A) *Ifngr1^−/−^* mice were infected with between 10^2^ and 10^6^ CFU of BCG, and animal survival (five animals per group) was monitored thereafter. (B) Splenic mycobacterial loads were determined on days 4, 15, 45, and 90 in infected *Ifngr1^−/−^* and *Ifngr^+/+^* mice; means of five animals per point are shown.

C57BL/6 *Ifngr1^−/−^* mice were unable to control BCG infection following intravenous injection with ≥ 10^2^ CFU (Figure 1A). Deficient animals survived 29 ± 2 weeks (mean ± SD) following challenge with 10^2^ CFU, whereas no mortality was observed in wild-type C57BL/6 control mice (*Ifngr1* ^+/+^) during 12 mo of follow-up, even after infection with the highest counts of CFU. The survival of deficient animals was inversely proportional to the size of the inoculum (Figure 1A), with mortality ranging from 29 wk with 10^2^ CFU to 12 wk with 10^6^ CFU. *Ifngr1^−/−^* mice challenged i.v. with 10^6^ CFU of BCG were unable to control the infection: the bacterial load in the spleen increased to 7.4 ± 0.3 (Log_10_ CFU) by 45 d after infection and exceeded 8 Log_10_ CFU in dying animals (Figure 1B). In *Ifngr1* ^+/+^ mice, the BCG burden was controlled, with a decrease in CFU observed by day 45, and CFU numbers falling to 5 Log_10_ by day 90. Granuloma formation was altered in BCG-infected *Ifngr1^−/−^* mice. Two weeks after infection, *Ifngr1^−/−^* mice had fewer and smaller granulomas in both the spleen and the liver than infected *Ifngr1* ^+/+^ control mice. Granulomas were mostly lymphoid, with no recruitment of epithelioid cells, whereas the granulomas observed in *Ifngr1* ^+/+^ BCG-infected mice contained mostly epithelioid cells and a few lymphocytes (unpublished data). By day 90 post-infection, *Ifngr1^−/−^* mice presented massive mycobacterial dissemination associated with large necrotising granulomas. In contrast, a very small number of small, well-delimited granulomas were observed in infected *Ifngr1* ^+/+^ control mice (unpublished data). These data are consistent with the absence of mature granulomas observed in patients entirely lacking IFN-γR1 [11]. Our results confirm that *Ifngr1^−/−^* mice are highly susceptible to *M. bovis* BCG infection, consistent with previous reports [27], and validate this model for the study of IFN-γR1 deficiency in humans.

### HSCT Restores Anti-Mycobacterial Immunity in *Ifngr1^−/−^* Mice

**Figure 2:**
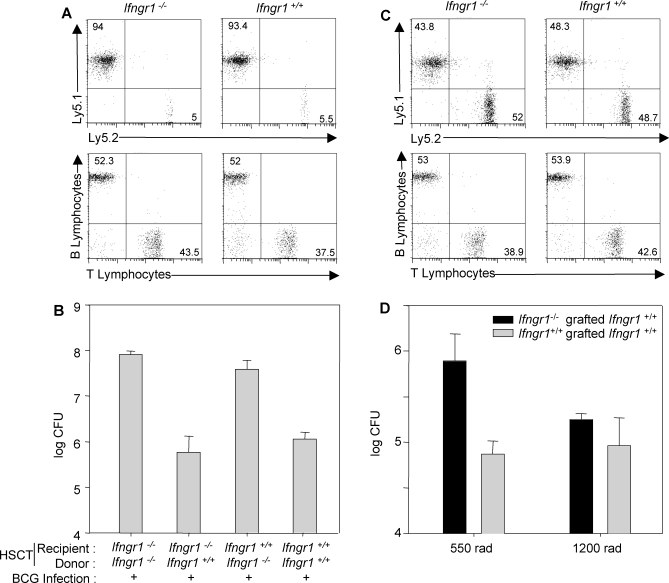
HSCT Restores Anti-Mycobacterial Immunity in *Ifngr1^−/−^* Mice (A) *Ifngr1^−/−^* and *Ifngr^+/+^* mice (five animals per group) expressing the Ly5.2 marker were subjected to HSCT with bone marrow from *Ifngr^+/+^* mice expressing the Ly5.1 marker, after intense irradiation (1,200 rads). Chimerism, assessed by determining the surface expression of Ly5.1 and Ly5.2 on lymphocytes, and peripheral reconstitution, assessed by determining the surface expression of TCRαβ and B220 markers on lymphocytes, were analysed by flow cytometry nine weeks after HSCT treatment. (B) *Ifngr1^−/−^* and *Ifngr^+/+^* mice were subjected to HSCT with bone marrow from *Ifngr1^−/−^* or *Ifngr^+/+^* mice. HSCT-treated mice were then infected with BCG and bacterial loads were determined 45 d later (five animals per group). (C) *Ifngr1^−/−^* and *Ifngr^+/+^* mice expressing the Ly5.2 marker were subjected to HSCT with bone marrow from *Ifngr^+/+^* mice expressing the Ly5.1 marker, after mild irradiation (550 rads). Chimerism and peripheral reconstitution were analysed by flow cytometry nine weeks after HSCT treatment. (D) *Ifngr1^−/−^* and *Ifngr^+/+^* mice were subjected to HSCT with bone marrow from *Ifngr^+/+^* mice after intense or mild irradiation, infected with BCG and bacterial load was determined 45 d later.

We subjected C57BL/6 *Ifngr1^−/−^* mice to HSCT with sex- and age-matched syngenic C57BL/6 *Ifngr1* ^+/+^ donors with an intensive total body irradiation conditioning regimen (1,200 rad). Several doses of bone marrow were tested and we found that 2 million cells was the dose most comparable to HSCT in humans. Leukocyte chimerism was complete, with low levels of residual autologous cells nine wk after treatment. Peripheral reconstitution was achieved, with the expected counts of lymphoid (T and B) and myeloid (macrophages and granulocytes) cells (Figure 2A, unpublished data). We then evaluated the ability of the recipient mice to control BCG infection. Ten weeks after HSCT, animals were challenged i.v. with 10^6^ CFU of BCG. They were killed 45 d later and BCG load in the spleen was determined. *Ifngr1^−/−^* animals transplanted with *Ifngr1^−/−^* bone marrow were unable to control BCG infection, like *Ifngr1* ^+/+^ animals receiving *Ifngr1^−/−^* bone marrow (Figure 2B). *Ifngr1^−/−^* animals receiving *Ifngr1* ^+/+^ bone marrow controlled the infection as efficiently as *Ifngr1* ^+/+^ mice receiving *Ifngr1* ^+/+^ bone marrow or mice with no graft (Figure 2B). HSCT recipients conditioned with a milder regimen (550 rads) displayed mixed chimerism, with only about 50% donor Ly5.1 lymphocytes (Figure 2C). The donor haematopoietic compartment had nonetheless restored BCG growth control 45 d after infection (Figure 2D). Thus, the restoration of IFN-γR1 expression in the haematopoietic compartment alone, even in only a fraction of the compartment, is sufficient to confer resistance to BCG infection. Mycobacterial disease in mice with IFN-γR1 deficiency therefore results from the specific absence of IFN-γR1 in the haematopoietic compartment, consistent with reported data for HSCT in human patients [12,13].

### Rejection of HSCT in *Ifngr1^−/−^* Mice Previously Infected with BCG

**Figure 3:**
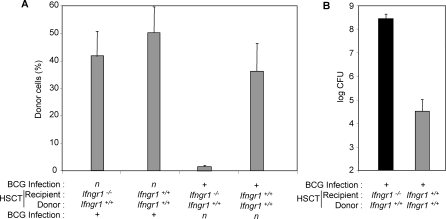
Rejection of the HSCT Graft in *Ifngr1^−/−^* Mice Previously Infected with BCG (A) Chimerism was determined by assessing the surface expression of Ly5.1 (donor cells) on lymphocytes, in *Ifngr1^−/−^* and *Ifngr^+/+^* mice treated by HSCT with bone marrow from *Ifngr^+/+^* mice, nine weeks post HSCT. *Ifngr1^−/−^* and *Ifngr^+/+^* mice were infected with BCG either before or after HSCT (five animals per group). (B) Bacterial loads were determined after the treatment of *Ifngr1^−/−^* and *Ifngr^+/+^* mice previously infected with BCG (three animals per group), by HSCT with bone marrow from *Ifngr^+/+^* mice.

All the IFN-γR1-deficient patients undergoing HSCT had a history of mycobacterial disease, as HSCT has not yet been attempted in an asymptomatic child [12–15]. We thus infected animals with BCG before HSCT. *Ifngr1^−/−^* and *Ifngr1* ^+/+^ mice were infected intravenously with 10^6^ CFU BCG and subjected to HSCT 40 d later, with sex- and age-matched syngenic *Ifngr1* ^+/+^ donors. Following intense irradiation (1,200 rads), both *Ifngr1^−/−^* and *Ifngr1* ^+/+^ BCG-infected mice displayed successful immune reconstitution with *Ifngr1* ^+/+^ bone marrow (unpublished data). However, with a milder conditioning regimen (550 rads) more closely mimicking the situation in human transplant patients in terms of the chimerism post HSCT [12–15], *Ifngr1^−/−^* mice infected with BCG before HSCT rejected the graft, with Ly5.1 lymphocytes from the donor phenotype accounting for less than 2% of the circulating cells, whereas about 50% chimerism was observed in the absence of BCG infection (Figure 3A). Serving as a control, *Ifngr1* ^+/+^ mice infected with BCG and subjected to HSCT with *Ifngr1* ^+/+^ donor marrow, under the same milder conditioning regimen, displayed successful engraftment and reconstitution. Bacterial loads, determined 11 wk after infection, reached 8.4 Log_10_ CFU per spleen in the *Ifngr1^−/−^* cohort, versus only 4.5 Log_10_ CFU in *Ifngr1* ^+/+^ mice (Figure 3B). Histological observations confirmed dissemination in the *Ifngr1^−/−^* cohort, contrasting with small, well-delimited granulomas in *Ifngr1* ^+/+^ animals (unpublished data). Thus, *Ifngr1^−/−^* mice infected with BCG reject HSCT, with features mimicking the graft rejection observed in IFN-γR1-deficient patients.

### HSCT Graft Rejection Is Associated with High Serum IFN-γ Levels

**Figure 4:**
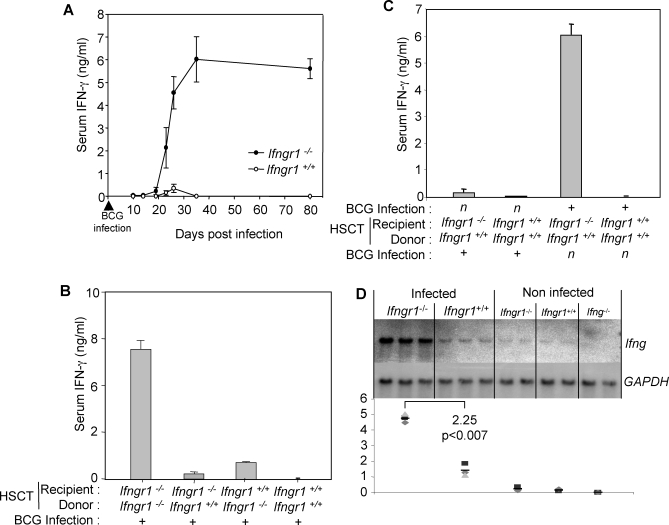
HSCT Graft Rejection Is Associated with High Serum IFN-γ Levels (A) IFN-γ levels were measured over time, in the serum of *Ifngr1^−/−^* and *Ifngr^+/+^* mice, after BCG infection (five animals per group). (B) IFN-γ levels were measured after the HSCT treatment of *Ifngr1^−/−^* and *Ifngr^+/+^* mice with either *Ifngr1^−/−^* or *Ifngr^+/+^* bone marrow (five animals per group). (C) IFN-γ levels were measured in *Ifngr1^−/−^* and *Ifngr^+/+^* mice treated by HSCT with *Ifngr^+/+^* bone marrow, and infected with BCG before or after HSCT (five animals per group). (D) Northern blot analysis of *Ifng* and *GAPDH* mRNA levels in the spleens of *Ifngr1^−/−^* and *Ifngr^+/+^* mice 30 d after BCG infection (three animals per group). Spleens were removed from animals, directly frozen in 1 ml of Trizol and stored at −80 °C for further preparation. Non-infected *Ifngr1^−/−^*, *Ifngr^+/+^*, and *Ifng^−/−^* mice were used as controls (two animals per group). Means of arbitrary values obtained after scanning were calculated and normalised with respect to the values obtained for GAPDH.

High serum IFN-γ concentration is a hallmark of human complete IFN-γR1 deficiency [35]. We thus monitored serum IFN-γ levels in BCG-infected *Ifngr1^−/−^* and *Ifngr1* ^+/+^ mice. IFN-γ was detected as early as 10 d post-infection and its concentration gradually increased towards a plateau at about 6 ng/ml within six wk of infection in *Ifngr1^−/−^* mice, confirming previous observations [36]. IFN-γ remained barely detectable in infected *Ifngr1* ^+/+^ mice (Figure 4A). Serum IFN-γ levels, determined 30 d after BCG infection in *Ifngr1^−/−^* and *Ifngr1* ^+/+^ mice previously subjected to HSCT, were found to correlate with the control of BCG infection. IFN-γ levels were high in *Ifngr1^−/−^* animals engrafted with *Ifngr1^−/−^* bone marrow and only marginally elevated in *Ifngr1* ^+/+^ animals engrafted with *Ifngr1^−/−^* bone marrow (Figure 4B). In these conditions IFN-γ levels were very similar to those in BCG-infected non-transplanted *Ifngr1^−/−^* mice (Figure 4A). IFN-γ was almost undetectable in both *Ifngr1^−/−^* mice engrafted with *Ifngr1* ^+/+^ bone marrow and *Ifngr1* ^+/+^ mice engrafted with *Ifngr1* ^+/+^ bone marrow, as for non-transplanted *Ifngr1* ^+/+^ animals (Figure 4B). Following HSCT with mild conditioning after M. bovis BCG infection, IFN-γ concentration seemed to be high only in the cohort of *Ifngr1^−/−^* animals infected at the time of cell transfer, and in such cases, HSCT was unsuccessful (Figures 3A and 4C). These results were confirmed by quantification of IFN-γ transcripts in the spleens of infected animals. Infected *Ifngr1^−/−^* mice contained significantly larger amounts of IFN-γ mRNA than infected *Ifngr1* ^+/+^ animals (Figure 4D). Basal levels of IFN-γ were detected in non-infected *Ifngr1^−/−^* and *Ifngr1* ^+/+^ mice. As expected, no IFN-γ mRNA was detected in *Ifng^−/−^* mice. The amounts of IFN-γ mRNA in infected *Ifngr1^−/−^* and *Ifngr1* ^+/+^ mice differed by a factor of 2.25 (*p* ≤ 0.007). Thus, serum IFN-γ concentration increased with BCG infection and was inversely correlated with HSCT engraftment in the murine model of IFN-γR1 deficiency.

### rAAV-IFN-γ Injection Promotes Graft Rejection

**Figure 5:**
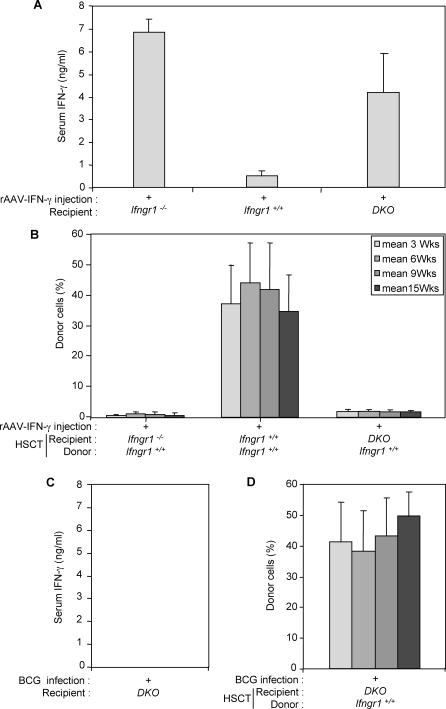
rAAV- IFN-γ Injection Promotes Graft Rejection (A) A rAAV- IFN-γ vector was designed, particles were produced and 5 × 10^10^ physical particles injected into the gastrocnemius of *Ifng^−/−^* mice. Serum IFN-γ levels were determined after rAAV- IFN-γ injection in *Ifngr1^−/−^*, *Ifngr1^+/+^* and DKO (*Ifngr1^−/−^*× *Ifng^−/−^*) mice (eight animals per group). (B) *Ifngr1^−/−^*, *Ifngr^+/+^*, and DKO mice were injected with rAAV-IFN-γ, and HSCT was performed with bone marrow from *Ifngr^+/+^* mice. The percentage chimerism over time was monitored by determining the surface expression of Ly5.1 (donor cells) on lymphocytes (eight animals per group). (C) Infected DKO mice showed no HSCT graft rejection. IFN-γ levels were measured following BCG infection, 30 d after infection (eight animals per group). (D) DKO mice were infected with BCG and the percentage chimerism over time was monitored by determining the surface expression of Ly5.1 (donor cells) on lymphocytes (eight animals per group).

We tested the hypothesis that high circulating IFN-γ levels are responsible for HSCT graft rejection, using a recombinant adeno-associated viral vector encoding IFN-γ (rAAV-IFN-γ). Following the intramuscular injection of 5 × 10^10^ rAAV-IFN-γ physical particles in non-infected *Ifngr1^−/−^* mice, IFN-γ was secreted ectopically in the muscle and accumulated in the serum (unpublished data), resulting on day 15 in serum IFN-γ levels ranging from 8 ng/ml to 11 ng/ml. Stable and sustained serum IFN-γ levels of 6 ng/ml to 7.5 ng/ml were obtained 30 d after rAAV-IFN-γ injection (Figure 5A), approaching those previously quantitated after BCG infection of *Ifngr1^−/−^* mice. HSCT with mild conditioning was thus performed in these mice on day 30 following rAAV-IFN-γ injection. In these conditions, HSCT graft rejection occurred in *Ifngr1^−/−^* recipients, with donor Ly5.1 lymphocytes accounting for only 0.46% ± 0.1% of cells (Figure 5B). In contrast, control *Ifngr1* ^+/+^ mice given intramuscular injections of rAAV-IFN-γ displayed successful engraftment and immune reconstitution. No detectable accumulation of IFN-γ occurred in *Ifngr1* ^+/+^ mice, probably because of the rapid clearance of IFN-γ by IFN-γR1-proficient cells. We detected minor increases in the serum levels of cytokines known to be involved in the IL12/23-IFN-γ circuit (IL12p40 in particular) in both naive and infected *Ifngr1^−/−^* and control *Ifngr1* ^+/+^ mice, at various stages of HSCT, with no repercussions outside this pathway (unpublished data). These data suggest that high circulating IFN-γ levels at the time of HSCT promote graft rejection in IFN-γR1-deficient mice, even in the absence of BCG infection.

### Infected DKO *Ifngr1^−/−^* × *Ifng^−/−^* Mice Show No Graft Rejection after HSCT

When infected with 10^6^ CFU of BCG, *Ifngr1^−/−^* × *Ifng^−/−^* double knock-out (DKO) mice were unable to control the infection and died within 12 wk of infection (unpublished data). As expected, IFN-γ was undetectable in serum (Figure 5C). Uninfected DKO mice displayed levels of chimerism after HSCT similar to those observed in *Ifngr1^−/−^* mice after total or mild conditioning (unpublished data). DKO mice controlled infection if HSCT with wild-type bone marrow was carried out before BCG infection (unpublished data). We also infected DKO mice with BCG and subjected them to HSCT, as described above. Bone marrow reconstitution was successful, and the level of chimerism obtained was similar to that in *Ifngr1* ^+/+^ and *Ifngr1^−/−^* HSCT-treated and BCG -infected mice (Figure 5D). Successful HSCT in these recipient mice ruled out a direct role for BCG infection, other than through IFN-γ induction, as the BCG burden in DKO mice was similar to that in *Ifngr1^−/−^* mice before cell transfer. We injected 5 × 10^10^ physical particles of rAAV-IFN-γ into DKO mice, resulting in high serum IFN-γ levels (4 ng/ml, 30 d after injection; Figure 5A). Following HSCT, engraftment was almost undetectable in these mice, with a mean of 2.4% (± 0.5%) donor Ly5.1 lymphocytes (Figure 5B). Thus, in the absence of IFN-γ, *Ifngr1^−/−^* mice displayed no rejection following HSCT, despite infection with BCG. In contrast, in the presence of high serum levels of IFN-γ, rejection occurred in these mice, even in the absence of BCG infection. In conclusion, these data clearly support the hypothesis that high circulating IFN-γ levels are necessary and sufficient for HSCT graft rejection in *Ifngr1^−/−^* mice.

### In Vivo Neutralisation of Circulating IFN-γ Allows HSCT Engraftment

**Figure 6:**
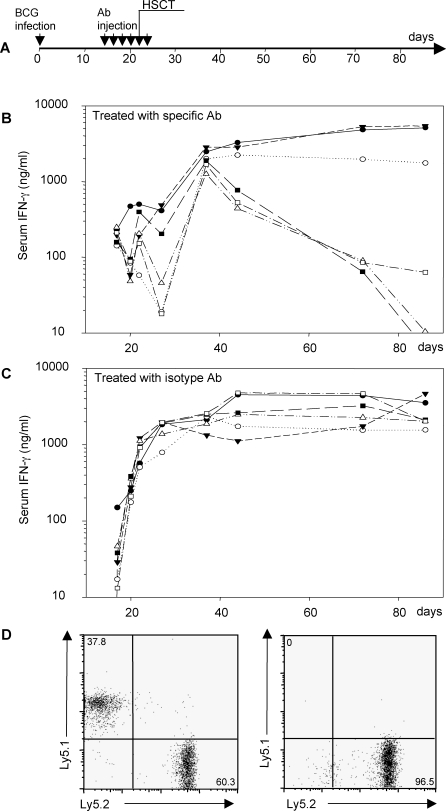
The Depletion of Circulating IFN-γ Is Sufficient to Allow HSCT Engraftment Groups of six *Ifng^−/−^* mice were infected with BCG and injected with a mixture of specific antibodies against IFN-γ and IL-12 or with a control isotype antibody. HSCT was carried out with bone marrow from *Ifngr^+/+^* mice and serum IFN-γ levels were monitored over time (A). Data are plotted individually for each animal treated with specific antibodies (B) and control isotype antibodies (C). The percentage chimerism was evaluated and representative FACS analyses are shown for engraftment (D), corresponding to three animals with low serum levels of IFN-γ (left) and rejection, corresponding to six animals with high serum levels of IFN-γ (right).

High serum IFN-γ concentration at the time of HSCT has a deleterious effect on engraftment. We tried to determine whether blood depletion of IFN-γ could render HSCT of infected *Ifngr1^−/−^* mice successful. A first series of experiments was performed with anti-IFN-γ antibody alone. In these conditions IFN-γ depletion was not achieved. We thus refined our protocol: animals were infected with BCG and received four intraperitoneal doses of anti-IFN-γ plus anti-IL-12 antibodies at two-day intervals, starting from day 14, before transplantation on day 23. Another antibody injection was administered on the day after HSCT. IFN-γ depletion was monitored by blood sampling and HSCT was performed with mild conditioning, mimicking that used for human patients (Figure 6A). The injection of specific antibodies into infected *Ifngr1^−/−^* mice kept serum IFN-γ levels below 0.5 ng/ml before cell transfer (Figure 6B). The injection of isotype control antibodies resulted in a serum IFN-γ concentration of 1.2 ng/ml at the time of HSCT, and ranging from 3 ng/ml to 5 ng/ml thereafter (Figure 6C). In three of the six animals treated with specific antibodies, serum IFN-γ levels did not exceed 3 ng/ml in the first week after HSCT and even decreased to 0.1 ng/ml by nine weeks post-HSCT (Figure 6B). Three other animals behaved like the isotype control-treated group, with serum IFN-γ levels remaining high throughout the experiment. HSCT outcome was found to be strictly correlated with serum IFN-γ concentration. Chimerism with bone marrow was observed in the three animals in which serum IFN-γ concentrations remained low (Figure 6D, left). In contrast, the graft was rejected in the three animals in which serum IFN-γ levels were not controlled; no donor cells were found in such animals (Figure 6D, right). Moreover, bacterial disease was cured in the three animals in which HSCT was successful (unpublished data). Our data therefore demonstrate that efficient IFN-γ depletion by antibody administration improves engraftment in *Ifngr1^−/−^* mice.

## Discussion

We have shown that the surface expression of functional IFN-γR1 in the haematopoietic compartment alone—actually in only about half of that compartment—is sufficient to protect mice against BCG infection. Similarly, Yap and Sher previously showed that IFN-γR1 expression in the haematopoietic compartment was sufficient to restore resistance to the intracellular macrophage-tropic bacterium Listeria monocytogenes in IFN-γR1-deficient mice [37]. However, they also showed that IFN-γR1 expression on both haematopoietic and non-haematopoietic cells was required to confer resistance to the macrophage-tropic intracellular protozoon Toxoplasma gondii [37]. Consistent with these findings, Dal Canto and Virgin showed that IFN-γ acted directly on both haematopoietic and non-haematopoietic cells during infection with the medial smooth muscle murine tropic γ-herpesvirus-68 [38]. Thus, despite the almost ubiquitous expression of IFN-γR1 on both haematopoietic and non-haematopoietic cells [39–41], BCG infection can be controlled—as attested by bacterial killing, granuloma structure, and animal survival—by the restricted action of IFN-γ on the haematopoietic compartment. Results obtained in our murine model are consistent with data from patients with complete IFN-γR1 deficiency undergoing HSCT [12–15] and demonstrate that susceptibility to BCG, and by extension to other mycobacteria, is a haematopoietic disease in persons with IFN-γR1 deficiency. In mice and humans, the extra-haematopoietic expression of IFN-γR1 is redundant for anti-mycobacterial protective immunity.

Likewise, in both mice and humans, the rate of HSCT graft rejection is also very high in individuals lacking IFN-γR1 and infected with mycobacteria. Moreover, high levels of circulating IFN-γ account for this high rate of rejection. *Ifngr1^−/−^* mice reject even syngeneic grafts from IFN-γR1-expressing mice, in all conditions resulting in high serum IFN-γ levels, including mycobacterial disease and intramuscular injections of rAAV-IFN-γ. We have shown that IFN-γ is necessary and sufficient for HSCT graft rejection. IFN-γ has already been shown to have a direct inhibitory effect on myeloid, erythroid, megakaryocyte, and multipotent colony formation in human cultures in vitro [42,43]. Moreover, in long-term bone marrow cultures—the in vitro assay best mimicking the complex interactions occurring in intact bone marrow—Selleri et al. showed that local IFN-γ overproduction by stromal cells inhibits haematopoiesis [44]. Two mechanisms seem to be responsible for the anti-haematopoietic effect of IFN-γ: the direct killing of stem cells and the inhibition of cell cycling [44,45]. The exposure of primitive human haematopoietic stem cells to IFN-γ in vitro increases Fas antigen expression, thereby rendering the cells more susceptible to apoptosis [46]. In vivo studies in mouse models have confirmed these results. Transgenic mice expressing multiple copies of the IFN-γ gene, leading to high levels of IFN-γ production in the bone marrow and thymus, display hypocellularity, and multiple alterations of the immune system [47]. There is also a strong correlation between the degree of heamatopoietic suppression and the level of IFN-γ in vitro [48,49]. Altogether, these observations most likely account for the detrimental effects of high levels of circulating IFN-γ in IFN-γR1-deficient mice and humans undergoing HSCT.

The negative impact of high IFN-γ levels, leading to HSCT graft rejection, has important clinical implications for the treatment of people with complete IFN-γR1 deficiency. Attempts could be made to decrease levels of circulating IFN-γ before HSCT. As recently shown, antibiotics may not eradicate M. fortuitum infection, but they do markedly decrease serum IFN-γ concentration before HSCT [15]. Prolonged treatment with multiple anti-mycobacterial antibiotics is therefore required, but is unlikely to decrease IFN-γ levels sufficiently and is unlikely to cure most of the patients [7]. In this study, we explored the use of specific antibodies to deplete the blood of IFN-γ. As anti-IFN-γ antibodies alone were not sufficient for IFN-γ depletion, we also used anti-IL-12p40 antibodies [50] to achieve efficient depletion of IFN-γ in the serum. Infected IFN-γR1-deficient mice treated in this way displayed engraftment and control of the mycobacterial infection after HSCT. Anti-IL-12 and anti-IFN-γ antibodies have already been used to treat human immune diseases, such as psoriasis and Crohn's disease [51–53]. The clinical use of these antibodies before HSCT is therefore feasible in IFN-γR1-deficient patients. Our study paves the way for improvements in transplantation conditions in people with IFN-γR1 deficiency, or with other diseases associated with elevated levels of circulating IFN-γ, such as IFN-γR2 deficiency [54]. We can also speculate that the use of such antibodies may also reduce the risks of HSCT rejection, improving engraftment, in other settings, such as HSCT from a haplo-identical or matched unrelated donor [16,55]. Although we did not observe any detrimental effect of rAAV-IFN-γ an HLA-identical setting in our mouse model, the depletion of IFN-γ in patients with conditions associated with high rates of graft rejection, such as HLA-II deficiency [56], or in other patients undergoing haplo-identical transplantations, might be beneficial. Further experimental studies in mice should be done before the corresponding clinical trials can be undertaken.

## Abbreviations

BCG: Mycobacterium bovis bacillus Calmette-Guérin
DKO: double knock-out
HSCT: haematopoietic stem cell transplantation
IFN-γ: Interferon-γ
IFN-γR1: Interferon-γ receptor 1
rAAV-IFN-γ: recombinant adeno-associated viral vector encoding IFN-γ
